# Exploring nanocomposites for controlling infectious microorganisms: charting the path forward in antimicrobial strategies

**DOI:** 10.3389/fphar.2023.1282073

**Published:** 2023-09-27

**Authors:** Harish Saravanan, Tarunkarthick Subramani, Shobana Rajaramon, Helma David, Anusree Sajeevan, Swathi Sujith, Adline Princy Solomon

**Affiliations:** Quorum Sensing Laboratory, Centre for Research in Infectious Diseases (CRID), School of Chemical and Biotechnology, SASTRA Deemed to be University, Thanjavur, India

**Keywords:** nanocomposites, antibacterial, silver, graphene, metals, polymers, phytochemicals

## Abstract

Nanocomposites, formed by combining a matrix (commonly polymer or ceramic) with nanofillers (nano-sized inclusions like nanoparticles or nanofibers), possess distinct attributes attributed to their composition. Their unique physicochemical properties and interaction capabilities with microbial cells position them as a promising avenue for infectious disease treatment. The escalating prevalence of multi-drug resistant bacteria intensifies the need for alternative solutions. Traditional approaches involve antimicrobial agents like antibiotics, antivirals, and antifungals, targeting specific microbial aspects. This review presents a comprehensive overview of diverse nanocomposite types and highlights the potential of tailored matrix and antibacterial agent selection within nanocomposites to enhance treatment efficacy and decrease antibiotic resistance risks. Challenges such as toxicity, safety, and scalability in clinical applications are also acknowledged. Ultimately, the convergence of nanotechnology and infectious disease research offers the prospect of enhanced therapeutic strategies, envisioning a future wherein advanced materials revolutionize the landscape of medical treatment.

## 1 Introduction

In the global scenario, the escalating emergence of antibiotic-resistant organisms presents a formidable global menace, jeopardizing lives worldwide (Bharadwaj et al., 2022). Infectious diseases, originating from a spectrum of pathogenic microorganisms, encompassing bacteria, viruses, fungi, and parasites, are inexorably linked with extended hospitalizations, culminating in elevated rates of morbidity and mortality (Purkayastha and Dahiya, 2012; Tanwar et al., 2014). Conventional strategies for managing infectious diseases invariably entail the utilization of antimicrobial agents, including novel antibiotics, antivirals, and antifungals, meticulously designed to target specific facets of microbial physiology. Nevertheless, these efforts have often yielded limited success due to the gain of resistance. Adding to the complexity of this challenge is the withdrawal of biopharmaceutical industries from the pursuit of novel antibiotics due to formidable economic and regulatory impediments, further accentuating the profound global healthcare predicament (Garg et al., 2022).

The relentless proliferation of multi-drug resistant bacteria (MDR) exacerbates this crisis, compelling the exploration of alternative therapeutic avenues. Consequently, scientists have embarked on the development of exceptionally potent drugs capable of combating a diverse array of bacterial systems, thereby making it exceedingly arduous for bacteria to develop resistance mechanisms. One promising strategy to address the shortcomings of traditional antibiotics is the integration of antimicrobial nanoscale materials (Birhanu et al., 2023).

A nanocomposite is a solid material characterized by multiple phases, with at least one phase exhibiting dimensions in one, two, or three directions that are smaller than 100 nm. Alternatively, it can have structures with nano-scale repeating distances between the various phases composing the material (Tasnim et al., 2017). They inherently possess distinctive physical and chemical attributes that render them highly advantageous for a myriad of applications, spanning from drug delivery to imaging and sensing (Petros and DeSimone, 2010). These nanoparticles encompass an expansive array of materials, including metals, metal oxides, polymers, and lipids, and can assume a diverse spectrum of shapes and properties. However, it is worth noting that nanoparticles have been identified as relatively toxic, thereby limiting their efficacy in medical applications. In contrast, nanocomposites (NCs) represent a class of materials comprising a matrix, often constituted by a polymer or ceramic, harmoniously integrated with nanofillers, such as nanoparticles or nanofibers. This combination of nanoparticles with polymers has been instrumental in mitigating the inherent toxicity of pure nanoparticles (Naskar and Kim, 2019; Omanović-Mikličanin et al., 2020). Notably, in recent times, metal nanoparticles (MNPs) and metal nanocomposites (MNCs) have demonstrated remarkable antimicrobial efficacy against drug-resistant bacteria, proficiently inhibiting the proliferation of these formidable strains. This positions nanocomposites as promising candidates for future antibacterial therapeutics, with the potential to augment treatment efficacy and curtail the emergence of antibiotic resistance. Crucially, the successful application of nanocomposites hinges on meticulous tailoring of the matrix and antibacterial agent (metal/compound) to optimize drug delivery systems (Alavi and Rai, 2019). The morphologies of these nanocomposites, such as their fabricated shape, surface charge, and zeta potential, determine their activity. Spherical and non-spherical nanoparticles enhance surface area, which enables close interaction with bacterial membranes, increased membrane deformation, and increased release of medicinal chemicals. Through physical interactions, puncturing, and increased electrochemical and surface catalytic reactivity, irregular edges of some non-spherical morphologies may help disturb bacterial cell walls. Additionally, highly reactive nanoparticle surfaces showed quick interactions with cellular membranes and exacerbated cell membrane damage (Sayed et al., 2022). The combination of minimal agglomeration and a low zeta potential could potentially enhance the absorption of nanoparticles by living organisms. This heightened uptake increases the likelihood of these nanoparticles entering cells, where they may have the potential to induce substantial damage to DNA and disrupt essential biological functions within these organisms (Jastrzębska et al., 2015).

Within this context, nanocomposites have emerged as a propitious frontier in the battle against infectious diseases, capitalizing on their distinctive physicochemical attributes and their ability to effectively engage with microbial cells. The subsequent sections of this review delve into an extensive spectrum of nanocomposites, encompassing metallic elements and phyto-molecules, with the aim of combatting infections and combatting pathogens that have demonstrated resistance to conventional drug therapies (Alavi and Rai, 2019).

## 2 Metal/metal-oxide nanocomposites

Metal nanocomposites offer a multifaceted approach to tackling pathogens. The nanocomposites of metals and metal oxides can be integrated into various matrices, such as polymers, ceramics, hydrogel matrices, etc., creating a synergistic effect that enhances their antimicrobial properties (Table 1). The intrinsic antimicrobial properties of metals stem from their capacity to produce reactive oxygen species (ROS) when they encounter microbial cells. Further, initiates oxidative stress, which interferes with vital cellular elements, ultimately causing the demise of the cells (Morones et al., 2005) (Kim et al., 2007) (Ozdal and Gurkok, 2022). Metal nanoparticles, due to their diminutive size, have the capability to infiltrate tbacterial cells and perturb their membranes, resulting in the release of internal constituents. Additionally, they can attach to bacterial DNA, impeding both transcription and replication processes. These nanoparticles also disrupt the electron transport chain, leading to a reduction in ATP production. In comparison to bare metals, metal nanoparticles, and conventional antimicrobial agents, metal nanocomposites offer distinct benefits. Metal nanocomposites encompass a notably elevated surface area-to-volume ratio and an adjustable character that facilitates precise management of factors like particle dimensions, morphology, and composition (Rajaramon et al., 2023b). Thus, enhances interaction with pathogens, thereby optimizing their effectiveness in combating microbial growth (Meng et al., 2018; Ribeiro et al., 2022) (Rai et al., 2009) (Gunduz et al., 2017).

**TABLE 1:** Biological effectiveness of diverse metal-based nanocomposites.

S.No	Material used	Organisms inhibited	Mechanism of action	Characteristics/Properties of material	Major outcomes	References
1.	Titanium dioxide-polytetrafluorethylene (TiO_2_-PTFE)	*E. coli, S. aureus*	• Synergistic effect	Sol-gel coating technique, improved hydrophilicity	• Antibacterial property	Zhang et al. (2019)
			• Antibacterial and anti-adhesion		• Corrosion resistance	
					• Improved biocompatibility	
2.	Graphene oxide-cuprous oxide (rGO-Cu_2_O)	*E. coli, S. aureus*	• Electrostatic interaction	Combination of rGO and Cu_2_O, improved dispersion	• Powerful long-lasting antibacterial capability	Díez-Pascual and Luceño-Sánchez (2021)
			• Sustained release of copper ions		• Enhanced ROS production	
			• ROS production			
3.	Chitosan/metal oxide polymeric	*E. coli, S. aureus*	• Antibacterial activity, distinction between mammalian and bacterial cells	Formation of NiO, MgO, CS-NiO, and CS-MgO	• Superior antibacterial efficacy	Taghizadeh et al. (2020)
					• Inhibition of bacterial pathogens	
4.	Ag-ZnO	*S. aureus*	• Synergistic antibacterial activity	Microwave-assisted synthesis, Ag and ZnO combination	• Pronounced antibacterial activity against *S. aureus*	Peng et al. (2017)
			• Sustained effect		• Lower MIC concentrations	
5.	Copper-zinc-manganese tri-metal oxide	*E. coli*	• Physical and chemical damage	Potent antibacterial activity, concentration-dependent	• Inhibition of *E.coli* growth	Alam et al. (2022)
			• Disruption of cell wall		• Breakdown of bacterial cells	
6.	Graphene/iron oxide	MRSA	• Physical and chemical damage	Potential for physical and chemical damage	• Inhibition of MRSA growth	Pan et al. (2016)
			• Disruption of cell wall		• Potential for antibacterial activity	

### 2.1 Silver-based nanocomposites

Silver nanocomposites (Ag-NC) offer an innovative strategy in the battle against microbial pathogens and present numerous advantages compared to traditional antimicrobial agents. The efficacy of silver (Ag) as an antimicrobial agent stems from its capacity to disrupt essential cellular mechanisms across a wide array of microorganisms. Through the controlled release of silver ions from these nanocomposites, a consistent and localized antimicrobial impact is achieved. The approach minimizes potential harm to host cells and diminishes the overall necessary dosage. Categorically, silver-based nanocomposites fall into distinct groups: i) Silver-polymer nanocomposites, ii) Silver-biopolymer nanocomposites, iii) Silver-inorganic compound nanocomposites, and iv) Silver (I) complexes.

#### 2.1.1 Silver-polymer nanocomposites

Nanoscale silver has been successfully synthesized and rendered stable within various polymer matrices, including polyacrylate (Mitra and Bhaumik, 2007), poly (amidoamine) (Zhang et al., 2008), polyaniline (Karim et al., 2007), poly (methyl methacrylate) (Kong and Jang, 2008), poly (ethylene oxide) (Chen et al., 2008) and numerous additional options. Incorporating silver into polymer nanocomposites has proven effective in achieving a regulated and extended discharge of silver nanoparticles (AgNPs). Consequently, this leads to an escalated antimicrobial efficacy when contrasted with standalone silver nanoparticles and silver (I) complexes (Wang et al., 2012). Polyrhodanine nanofibers embedded with silver nanoparticles were fabricated using rhodanine, showcasing potent antimicrobial effects against *E. coli*, *S. aureus*, and *C. albicans* (Dallas et al., 2011). The effectiveness of polyacrylic acid/silver nanocomposite hydrogels against a set of standard bacterial strains including *C. albicans*, *S. aureus*, *P. aeruginosa*, and *E. coli*, as well as five clinical bacterial strains encompassing *S. aureus*, *S. epidermidis*, *P. aeruginosa*, *Acinetobacter baumannii*, and *Klebsiella pneumonia*, was evaluated. These hydrogels demonstrated strong inhibition of *E. coli* and *S. aureus*, while displaying comparatively less potency against yeast. (Wei et al., 2016).

#### 2.1.2 Silver-biopolymer nanocomposites

The combined impact of chitosan and silver nanoparticles has exhibited the ability to hinder a range of gram-positive bacteria. Results from microbial activity assessments highlighted that AgNP-chitosan spheres treated with 50% NaOH yielded the largest inhibition zones against *S. aureus* and *E. coli*, measuring 19.5 mm and 18 mm in diameter, respectively (Mirda et al., 2021). The inhibitory effect of chitosan-silver nanocomposites was observed against *S. aureus, E. coli, P. aeruginosa,* and *S. enterica*. Upon exposure to chitosan-silver nanoparticles, notable alterations in the morphology of *S. aureus* were observed, attributed to compromised cell wall integrity following incubation (Potara et al., 2011; Kaur et al., 2013; Kumar-Krishnan et al., 2015). Ag-infused chitosan nanocomposites, in conjunction with the fungicide Antracol, were employed to address Phytophthora capsi-induced Phytophthora blight in pepper plants. The Ag-chitosan/Antracol nanocomposite displayed noteworthy fungicidal efficacy, surpassing the individual impact of each constituent component (Le et al., 2019). An extensive investigation targeting *B. subtilis*, *S. aureus*, and *K. pneumonia* was conducted using Cellulose/Ag nanocomposites. The incorporation of the metal, specifically silver (Ag), was thoroughly explored, with the bacterial cellulose/Ag (BC-Ag0.05) nanocomposite demonstrating the most pronounced antibacterial properties. The outcome implies a direct correlation between the concentration of silver and the antimicrobial effectiveness of the nanocomposites. The noteworthy ability of bacterial cellulose nanocomposites to impede the proliferation of diverse bacteria and fungi, encompassing *E. coli*, *S. aureus*, *B. subtilis*, and *C. albicans*, underscores their potential as versatile materials with promising prospects for medical applications (Shao et al., 2015). Even at minimal concentrations of 5.0 × 10^−4^ wt%, the presence of AgNPs within the cellulosic fibers renders these nanocomposites proficient as antibacterial materials (Pinto et al., 2009).

Composite films composed of pullulan and AgNPs were assessed for their effectiveness against *A.niger*. The integration of AgNPs into the pullulan matrix led to the disintegration of *A. niger* spores. Leveraging pullulan as a biocompatible foundation enhances the applicability of these nanocomposites in the creation of antifungal packaging materials. The Ag/pullulan nanocomposites demonstrated notable potency in hindering *A.niger* sporulation, resulting in diminished fungal viability (Pinto et al., 2013).

#### 2.1.3 Silver-inorganic compound nanocomposites

Silicon (Si) and its nanomaterial derivatives have been acknowledged for their capacity to aid plants in alleviating the adverse effects of both abiotic and biotic stressors. A silver/silicon dioxide nanocomposite (Ag/SiO_2_NC), synthesized utilizing the *E. coli* free-cell supernatant, was harnessed as an antifungal agent to counteract *B. cinerea* infection in faba bean (*Vicia faba* L.). The Ag/SiO_2_NC, with positively charged silver nanoparticles, demonstrated potent *in-vitro* antifungal potency, displaying a minimal inhibitory concentration (MIC) of 40 ppm. The *in-vitro* efficacy of the fabricated Ag/SiO_2_NC against *B. cinerea* underscores its disease-control potential. Corroborating these findings, *in vivo* tests demonstrated that plants subjected to Ag/SiO_2_NC treatment displayed increased resistance to fungal infection. The enhancement was accompanied by biochemical and ultrastructural modifications that are closely associated with the activation of plant defense mechanisms (Baka and El-Zahed, 2022). The antifungal capabilities of ZrO_2_-Ag_2_O nanoparticles, produced through the sol-gel technique, were investigated against diverse *Candida species*, encompassing *Candida albicans*, *Candida dubliniensis*, *Candida glabrata*, and *Candida tropicalis*. Outcomes from the assessment of antifungal activity demonstrated the efficacy of ZrO_2_-Ag_2_O NPs against *Candida* species. Moreover, a viability analysis on human mononuclear cells indicated that neither time nor concentration significantly impacted cell viability. These encouraging findings contribute to the advancement of potential alternative therapeutic avenues for addressing fungal infections in humans (Ayanwale et al., 2021). The bactericidal efficacy of Ag-ZnO nanocomposites was investigated using recombinant green fluorescent protein-expressing antibiotic-resistant strains of *E. coli* and *S. aureus*. *S. aureus* and *E. coli* were found to have minimum inhibitory concentrations (MICs) of 60 g/mL and 550 g/mL, respectively (Matai et al., 2014).

#### 2.1.4 Silver (I) complexes

Silver complexes have exhibited antimicrobial attributes against an extensive spectrum of bacteria and fungi. They have been established as potent anti-infective agents with minimal cytotoxic effects. Notably, silver (I) complexes demonstrate a distinct antimicrobial range compared to the ligand and the hydrated silver (I) ion. A well-known example is the use of the silver sulfadiazine complex for managing burns and wounds. Moreover, these silver complexes are frequently employed as additives in diverse polymer and biopolymer composites to address infectious ailments (Wang et al., 2012).

### 2.2 Chitosan-metal based nanocomposites

**FIGURE 1:**
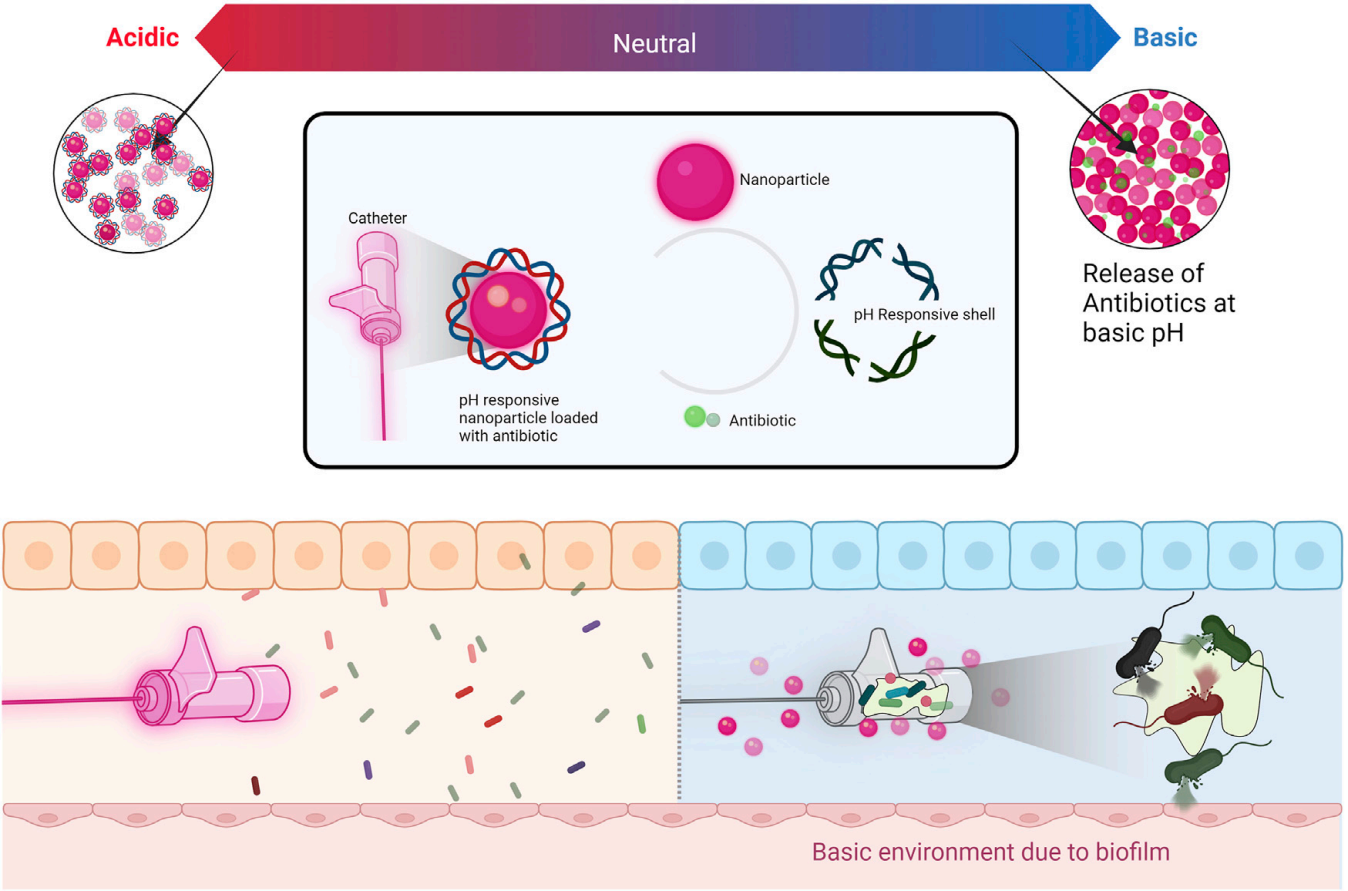
pH response release of antibiotics from nanocomposite coated catheter.

Chitosan ranks as the second most prevalent polysaccharide found in nature. Its interaction with the negatively charged bacterial cell wall leads to cell disruption, while also hindering DNA replication and selectively binding to trace elements, consequently impeding toxin production. These mechanisms collectively contribute to the capacity of chitosan to restrain the proliferation of microorganisms (Cuero et al., 1991; Divya et al., 2017). The antibacterial potential of Chitosan/metal oxide (CS-MO) polymeric nanocomposites was assessed against *E. coli* and *S. aureus*. When compared to utilizing chitosan or metal oxides separately, these CS-MO nanocomposites demonstrated increased antibacterial efficacy. The superior antibacterial effectiveness of the chitosan/nickel oxide (CS-NiO) nanocomposite, which reduced *S. aureus* and *E. coli* viabilities to 2–8 percent after a 12-h incubation period, was particularly remarkable. Based on these outcomes, it was concluded that CS/NiO nanocomposites hold the potential to act as antibacterial agents against harmful bacterial pathogens. The polymeric materials based on metal oxides can effectively differentiate between mammalian and bacterial cells, offering prolonged antibacterial growth suppression. The particle sizes observed were 18.3 nm for CS-NiO and 25.5 nm for CS-MgO. All samples exhibited antibacterial activity against both *E. coli* and *S. aureus* strains, with a stronger effect on *S. aureus* compared to *E. coli*. When treated with 15 mg/mL of CS-NiO, inhibition rates reached 98% for *S. aureus* and 92.3% for *E. coli* cells. Additionally, the photocatalytic degradation of *E. coli* and *S. aureus* was investigated using ZnO, AgCl, and AgCl/ZnO nanocomposite-incorporated chitosan hydrogel beads. The process involved the breakdown of these gram-negative and positive bacteria using a chitosan/AgCl/ZnO (CS/AgCl/ZnO) nanocomposite hydrogel beads system as a photocatalyst, specifically under visible light irradiation (Taghizadeh et al., 2020). The antimicrobial potential of a nanocomposite containing copper-zinc-manganese tri-metal oxide was investigated against *E. coli*. The range of inhibition zones varied between 12 mm and 16 mm, with the smallest and largest observed at concentrations of 25 μg/mL and 100 μg/mL, respectively. In the concentrations of 50 μg/mL and 75 μg/mL, the inhibition zones were measured at 13.5 mm and 14.6 mm, respectively. The observed result was attributed to the direct interaction occurring between the lipopolysaccharide molecules present on the bacterial cell wall and the engineered nanoparticles. The interaction caused disruption to the cell wall, ultimately resulting in its disintegration and subsequent death of the bacteria (Alam et al., 2022). The synthesis of chitosan-ZnO nanocomposites differs from their TiO_2_/chitosan counterparts due to the possible release of aqueous Zn^2+^ by ZnO nanoparticles. The phenomenon can induce changes in nanoparticle morphology and effectiveness. To date, there is no documented evidence of varying antimicrobial efficiencies of chitosan-ZnO nanocomposites against Gram-negative and Gram-positive bacteria. Additionally, hybrid nanocomposites of chitosan-ZnO and silver exhibit inhibitory effects. Chitosan when combined with metronidazole and hydroxyapatite has exhibited pH response drug release. The release amount of the combination was higher in the acidic pH than that of neutral environment. The controlled release might be due to the electrostatic repulsion force between the metronidazole and chitosan in acidic medium (Heragh et al., 2022) (Figure 1). Research investigating films composed of chitosan-Ag nanoparticles suggests a stronger antibacterial effect against Gram-negative bacteria compared to their Gram-positive counterparts. Therefore, this attribute could provide benefits when integrated with additional nanoparticles that exhibit remarkable antimicrobial potential against Gram-positive bacteria (Babaei-Ghazvini et al., 2021).

### 2.3 Titanium-based nanocomposites

**FIGURE 2:**
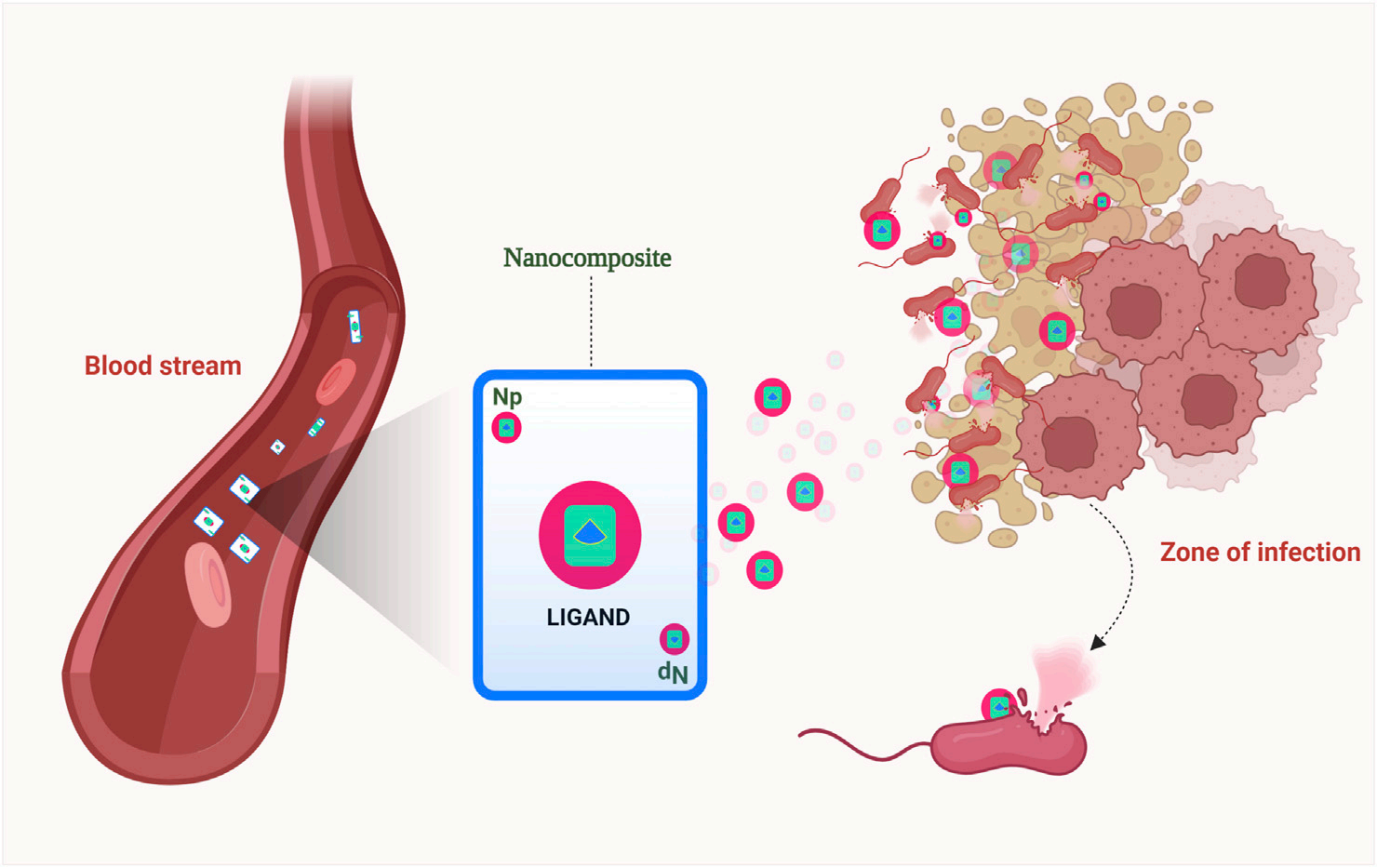
Metal oxide nanocarriers in targeted drug delivery systems.

When exposed to UV light in the presence of water and oxygen, TiO_2_ nanoparticles exhibit photocatalytic characteristics. The situation triggers the generation of reactive oxygen species, leading to the emergence of hydroxyl and superoxide free radicals. These radicals then engage with cellular macromolecules within the cell or interact with the phospholipids present in the cell membrane, resulting in a significant disruption of both cell membrane integrity and DNA (Babaei-Ghazvini et al., 2021). Zinc oxide (ZnO) nanoparticles, akin to titanium dioxide (TiO_2_) nanoparticles, also display photocatalytic properties. However, ZnO nanoparticles offer improved biocompatibility and stronger UV light absorption compared to TiO_2_. Regarding the generation of reactive oxygen species (ROS), ZnO nanoparticles share antimicrobial capabilities with TiO_2_ nanoparticles. Notably, ZnO photocatalytic processes under UV irradiation have been found to potentially produce hydrogen peroxide (Barnes et al., 2013).Titanium compounds are widely utilized in dental implants and possess a significant capacity for generating reactive oxygen species (Basiron et al., 2019). The viability of *S. aureus* diminished due to the photoactivation of TiO_2_ released from TiO_2_ encapsulated within polylactic (PLA) microspheres (Campardelli et al., 2013). The combination of titanium dioxide-polytetrafluoroethylene nanocomposite (TiO_2_-PTFE) exhibited inhibitory effects against *E. coli* and *S. aureus*. The antibacterial and anti-adhesion properties against these bacterial strains were attributed to the synergistic interplay between TiO_2_ and PTFE. Furthermore, both TiO_2_ and PTFE coatings demonstrated exceptional corrosion resistance and enhanced biocompatibility (Zhang et al., 2019). Nanoscale titanium powders doped with silver exhibited inhibitory effects against both *E. coli* and *Bacillus sp.* (Lavrynenko et al., 2022)*.* Within the domain of drug delivery systems (Figure 2), titanium dioxide nanoparticles play a critical role. Benefiting from its considerable surface area, compact size, robust stability, and cost-effectiveness, TiO_2_ stands out as an exemplary selection for nanocarriers in drug delivery systems. The integration of TiO_2_ nanocarriers streamlines drug administration at diminished doses, leading to a prolonged presence within the vascular system and intensifying their therapeutic efficacy (Saha et al., 2023). A drug delivery system employing biocompatible nanoparticles was developed by encapsulating ciprofloxacin within TiO_2_ nanocomposites. The system was harnessed as a therapeutic solution to combat multidrug-resistant *E. coli* strains causing infectious diseases in livestock (Zafar et al., 2022). Studies focusing on TiO_2_, and chitosan nanocomposites have demonstrated higher levels of inhibition when compared to pure chitosan films, primarily attributed to the supplementary inhibitory impact of TiO_2_ nanoparticles. Notably, TiO_2_ nanoparticles exhibit a more pronounced inhibitory effect when subjected to UV exposure as opposed to white light or storage in darkness. Furthermore, research findings suggest that TiO_2_ nanocomposites generally display enhanced antimicrobial efficacy against Gram-positive bacteria when contrasted with their impact on Gram-negative bacteria (Babaei-Ghazvini et al., 2021).

### 2.4 Graphene-based nanocomposites

**FIGURE 3:**
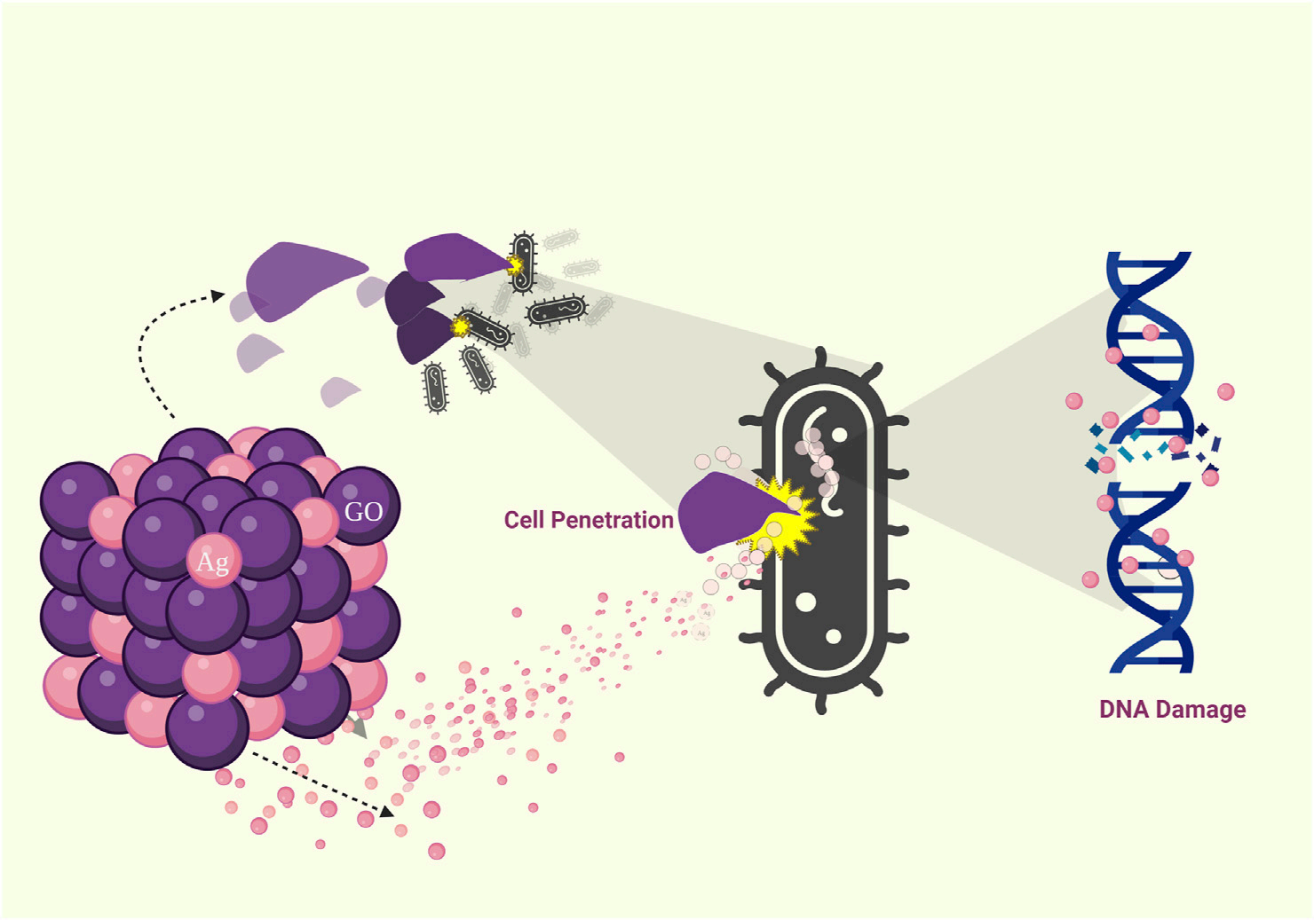
Visual representation illustrating the various modes of antimicrobial action of Graphene Oxide (GO): Cell penetration, Reactive Oxygen Species (ROS) generation, DNA damage, and cell death.

**FIGURE 4:**
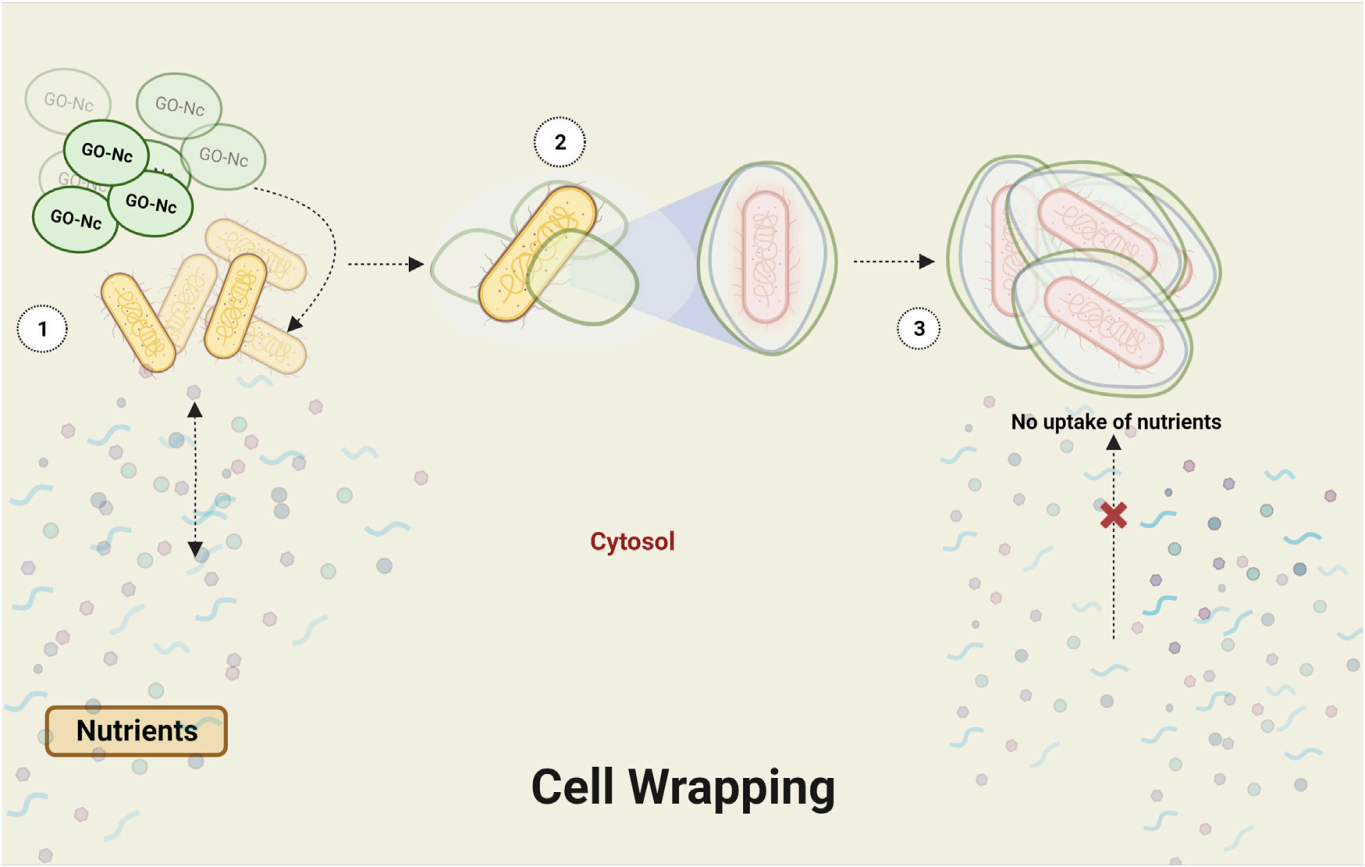
Eliciting the bacterial wrapping effect through graphene oxide (GO) interaction.

Graphene oxide (GO), a single-atom-thick lattice of carbon atoms, exhibits exceptional properties. It is being explored for diverse applications such as nanoelectronics, conductive films, supercapacitors, biosensors, and nanomedicine and has recently gained attention for its antibacterial properties. While challenges remain, GO’s unique attributes hold promise for innovative antibacterial applications. Derivatives of graphene found in nanocomposites include graphene oxide, reduced graphene oxide (rGO), and graphene quantum dots (GQDs) (Díez-Pascual and Luceño-Sánchez, 2021) (Table 2). Apparently, graphene oxide in nano size has sharp edges and appears like nanosheets, which can penetrate and tear the cell wall causing cytoplasmic leakage (Rajaramon et al., 2023a) (Figure 3). The processes through which Gram-positive and Gram-negative bacteria are physically damaged by GO differ fundamentally. Gram-positive bacteria lack an outer coat, making them more vulnerable to GO and rGO. They are inactivated by a process known as “bacterial wrapping,” in which GO interacts with the gram-positive bacteria and physically wraps around them, disrupting their membranes and causing cell death (Figure 4). Gram-negative bacteria, on the other hand, have an outer membrane layer that acts as a protective barrier against GO. The inactivation of Gram-negative bacteria by GO primarily occurs through physical contact, causing damage to the bacterial cell membrane (Pulingam et al., 2019). The chemical mechanisms of GO interaction with bacteria include the self-killing effect and oxidative stress with or without ROS production (Mohammed et al., 2020). Research has also indicated that GO has the potential to disturb bacterial cell membranes, resulting in the release of contents and harm to the membrane structure.

**TABLE 2:** Biological effectiveness of different graphene-oxide-based nanocomposites.

S.No	Material used	Organisms inhibited	Mechanism of action	Characteristics/Properties of material	Major outcomes	References
1.	rGO-Cu_2_O	*E. coli, S. aureus*	• Electrostatic interaction	High surface area, long-lasting antibacterial activity	• Demonstrated effective inhibition	Díez-Pascual and Luceño-Sánchez (2021)
			• Sustained release of cu ions			
2.	GO, rGO	*E. coli, P. aeruginosa, S. aureus*	• Physical damage to the cell membrane	Large surface area, demonstrated toxicity	• Shown toxicity against bacteria	Zou et al. (2016)
			• Oxidative stress			
3.	GO-CuO	Gram-positive and Gram-negative bacteria	• Synergistic contribution of GO and CuO to the polymer matrix	Enhanced antimicrobial properties, sustained release of ions	• Improved antimicrobial properties	Xie et al. (2020)
4.	Graphene quantum dots	*S. aureus*	• Bactericidal effects	Small size, strong inhibition against multiple organisms	• Demonstrated bactericidal effects	D’Amora et al. (2023)
		*S. agalactiae, MRSA*	• Disruption of cell membranes			
		*P. aeruginosa*				
5.	rGO/-Mn_2_O_3_, Mn_2_O_3_	*P. fluorescens, E. coli, S. aureus, B. subtilis, P. aeruginosa*	• Physical damage to cell membrane	Interaction with bacterial cells, high toxicity	• Shown high toxicity against multiple organisms	Selim et al. (2023)
			• High toxicity			
6.	rGO-AgO	*S. aureus, C. albicans, E. coli*	• Physical damage to cell membrane	Effective antimicrobial activity, high inhibition	• Demonstrated superior antimicrobial capabilities	Elbasuney et al. (2023)
			• Protein leakage			
7.	SF@GOC	*E. coli, P. aeruginosa, S. aureus*	• Aggregation-induced distress in cell membrane	Strong inhibition against *C. albicans*	• Shown strong inhibition against multiple organisms	Fatima et al. (2021)
8.	CdO@Gr	*E. coli, S. aureus, S. typhimurium, E. faecalis, C. albicans*	• Green synthesis	Synthesized from onion extract, inhibitory effects	• Demonstrated inhibitory effects against multiple organisms	Hassanien et al. (2019)
			• Inhibitory effects			
9.	Ag-GO2% (CMC/SA/Ag-GO2%)	*E. coli, S. aureus*	• Synergistic reinforcement	Antibacterial activity against *E. coli*, inhibition against *S. aureus*	• Shown better activity against *E. coli* than *S. aureus*	Das et al. (2023)
			• Inhibition of growth			
10.	rGO–ZnO	*E*. *coli*	• Relatively higher cytotoxicity than streptomycin antibiotics	High cytotoxicity	• Shown higher cytotoxicity against *E. coli*	Rajaura et al. (2017)
11.	Magnetic samples (Mag/Cr, Mag/Cr/Ce, Mag/Cr/rGO,Mag/Cr/Ce/rGO)	*S. aureus*	• Magnetic properties	Magnetic properties, significant antibacterial activity	• Demonstrated significant antibacterial activity	Sadeghi Rad et al. (2021)
			• Significant antibacterial activity			
12.	Ag nanoparticle decorated graphene	*C. albicans, L.acidophilus, S.mutans, Aggregatibacter actinomycetemcomitans*	• Antimicrobial effects	Antimicrobial effects	• Demonstrated antimicrobial activities against multiple pathogens	Peng et al. (2017)

Meanwhile, an innovative approach was employed to design a stable graphene-oxide cuprous oxide (rGO- Cu_2_O) nanocomposite, utilizing the electrostatic interaction and unique electronic transition between rGO and Cu_2_O. The design showcased remarkable antibacterial capabilities and persistent effectiveness. The results of antibacterial testing demonstrated that the rGO-CuO nanocomposites exhibited potent and enduring antibacterial potential against *E. coli* and *S. aureus*. Through stability testing, copper ion release analysis, and ROS detection, the enhanced antibacterial mechanisms of rGO-Cu_2_O nanocomposites were systematically unveiled. These mechanisms were attributed to a synergistic effect involving the sustained release of copper ions, increased production of reactive oxygen species, and excellent dispersion of the rGO-Cu_2_O nanocomposites. Additional proposed mechanisms of action encompassed the prolonged release of copper ions due to cellular encapsulation, damage to bacterial cell membranes caused by the presence of sharp edges on rGO nanosheets, and the generation of ROS (Yang et al., 2019). In comparison to rGO-Cu_2_O, GO-CuO has shown a significant effect against both Gram-positive and Gram-negative bacteria. However, the effectiveness is particularly pronounced against Gram-positive bacteria (Xie et al., 2020). Apart from the substantial inhibition observed against *S. aureus* and *S. agalactiae*, biomaterials utilizing graphene quantum dots exhibited bactericidal properties against methicillin-resistant *E. coli* and *P. aeruginosa* (D’Amora et al., 2023).

The α-Mn_2_O_3_-functionalized rGO nanorods demonstrated remarkable resistance against a range of aerobic and anaerobic microorganisms such as *Kocuria rhizophila*, *B. subtilis*, *P. fluorescens, P. aeruginosa*, *S. aureus*, *A. brasiliensis*, *C. albicans*, and *Desulfovibrio halophilus* (Selim et al., 2023). The nanocomposite of rGO/α-Mn_2_O_3_ exhibited greater resistance to Gram-positive microorganisms compared to Gram-negative ones. The study underscored the distinction in antimicrobial mechanisms when targeting various aerobic and anaerobic microbes. The suggested mechanism for the bactericidal activity involved the wrapping effect resulting from the covalent interaction between rGO/α-Mn_2_O_3_, leading to mechanical stress and physical damage to bacterial cell membranes caused by the sharp edges of rGO. When comparing multiple rGO nanocomposites (AgO, NiO, ZnO) in terms of their antibacterial and antibiofilm effectiveness, rGO-AgO displayed significant antibiofilm activity against *S. aureus* (91.72%), *C. albicans* (91.17%), and *E. coli* (90.36%). After subjecting *S. aureus* to treatment with rGO-AgO, scanning electron microscopy revealed noticeable changes in morphology, including complete lysis of the outer surface and deformation of bacterial cells. The release of cellular protein from *S. aureus* increased proportionally with the concentration of rGO-AgO, rGO-NiO, and rGO-ZnO nanocomposites, shedding light on the antibacterial mechanism of action (Elbasuney et al., 2023). The nanocomposite GO@CS/ZnO, comprising zinc oxide-decorated chitosan and graphene oxide, exhibited both minimum inhibitory concentration and minimum bacterial concentration (MBC) effects against both *S. aureus* and *E. coli.* Synergistic enhancement of carboxymethyl-cellulose (CMC) and sodium alginate nanocomposite films was observed through the incorporation of graphene oxide and nano silver, leading to the inhibition of *E. coli* and *S. aureus* growth. The CMC/SA/Ag-GO2% nanocomposite displayed the highest zone of inhibition, measuring 0.70 mm against *E. coli* and 1.00 mm against *S. aureus*. The finding implies that the CMC/SA/Ag-GO2% nanocomposite exhibited superior antibacterial activity against *E. coli* compared to *S. aureus* (Das et al., 2023). Utilizing onion extract, a green synthesis method was employed to create a nanocomposite of Cadmium oxide-graphene (CdO@Gr), which exhibited inhibitory properties against various microorganisms including *E. coli*, *S. aureus*, *S. typhimurium*, *E. faecalis*, and *C. albicans* (Hassanien et al., 2019)*.* The graphene nanocomposite adorned with AgNPs displays antimicrobial properties against oral pathogens, including *C. albicans*, *Lactobacillus acidophilus*, *Streptococcus mutans*, and *Aggregatibacter actinomycetemcomitans* (Peng et al., 2017). The commonly accepted mechanism for the antibacterial and antifungal effects of GO involves the aggregation of its nanosheets. This aggregation induces disruption in the cell membrane, leading to a decrease in cell membrane potential and subsequent leakage from the cell (Fatima et al., 2021). Moreover, an innovative method involving the synthesis of magnetic samples using transition metals (Mag/Cr, Mag/Cr/Ce, Mag/Cr/rGO, and Mag/Cr/Ce/rGO) was assessed for their ability to generate reactive oxygen species to achieve antimicrobial effects against *S. aureus* (Sadeghi Rad et al., 2021). The Mag/Cr/Ce/rGO combination demonstrated enhanced efficacy, with the potential mechanism of action attributed to oxidative stress caused by nanoparticles and concurrent physical damage. This correlated well with mechanisms previously proposed by other researchers. In subcutaneous abscesses, a synergistic approach involving reduced graphene oxide-iron oxide nanoparticles (rGO-IONP) was found to effectively inflict both physical and chemical damage on methicillin-resistant *S. aureus* (MRSA). The nanocomposite system of rGO–IONP was synthesized to capitalize on the synergistic effects that result in the simultaneous physical and chemical harm to MRSA. The rGO efficiently absorbs and converts near-infrared energy into localized heat, leading to physical damage to the bacteria. Concurrently, the IONP catalyzes the breakdown of H_2_O_2_, which is significantly elevated due to local inflammation, producing highly reactive •OH radicals through Fenton reactions. This chemical process further contributes to the damage inflicted on MRSA (Pan et al., 2016).

### 2.6 Multi-walled carbon nanotubes based nanocomposites

The combined utilization of multi-walled carbon nanotubes (MWCNTs) and silver nanoparticles has been extensively explored for their antibacterial properties. Numerous studies have demonstrated the capacity of MWCNTs to induce severe membrane damage and cell death in various pathogens, underscoring their potential as antimicrobial agents. The incorporation of silver nanoparticles onto MWCNTs has shown as a valuable substance with antibacterial efficacy across diverse medical applications and water disinfection strategies aimed at curtailing the spread of infectious diseases. Ag-MWCNTs have exhibited broad-spectrum activity against a range of Gram-positive and Gram-negative bacteria, including *P. aeruginosa, E. coli, B. subtilis, S. aureus, C. albicans, A. niger, Methylobacterium spp.,* and *Sphingomonas spp.* (Cao et al., 2015). The fabricated nanocomposite has demonstrated elevated antimicrobial efficacy against Gram-positive bacteria in comparison to Gram-negative bacteria. The effectiveness can be attributed to the nanocomposite’s interaction with sulfur-based molecules found on both the cell membrane and within the microbial cell itself. As a result of this interaction, metabolic processes are disrupted, proteins undergo denaturation, and damage is inflicted within the microbial system. Yakdoumi et al. devised three nanocomposite films, namely PLA/MWCNTs, PLA/PDA-MWCNTs, and PLA/TiO_2_-PDA-MWCNTs, to assess their bactericidal capabilities. The PLA film alone did not exhibit any inherent antimicrobial activity against *S. aureus, P. aeruginosa,* and *S. epidermidis*. However, when combined with MWCNTs or functionalized MWCNTs, the PLA significantly augmented its antibacterial property (Yakdoumi et al., 2022).

Moreover, it has been observed that the inclusion of MWCNTs alongside diverse nanoparticles like ZnO, Ag, and Hydroxyapatite results in enhanced antimicrobial effectiveness. These nanocomposites exhibited noteworthy antimicrobial and antibiofilm activities against *S. aureus, B. subtilis, P. aeruginosa, E. coli,* and *C. albicans*. It was noteworthy to observe the substantial escalation in antimicrobial activity at low concentrations of MWCNTs in combination with ZnO and Ag (David et al., 2021).

## 3 Polymer-based nanocomposites

Materials with a polymer matrix and nanofillers acting as reinforcing agents are called polymeric nanocomposites. The main reasons for promoting polymeric nanocomposites are their low cost, ease of shaping into desired shapes, high surface area, low molecular weight, biocompatibility, quick degradation, and water solubility (Bharadwaz and Jayasuriya, 2020). Polymeric nanocomposites come in two varieties: synthetic and natural polymers. Wool, silk, cellulose, DNA, and proteins are examples of natural polymers, whereas polyethylene, Teflon, nylon, epoxy, and other synthetic polymers are natural polymers. Natural polymers are extracted from plants and animals, followed by purification, and modification. The preparation of polymer matrix nanocomposites is carried out by incorporating the nanoparticles in the matrix made of polymer. These nanocomposites are synthesized in various methods such as *in situ* synthesis, solution mixing, melt intercalation, electrospinning (Darwish et al., 2022), one-pot synthesis (Cao et al., 2014), self-assembly, inter-matrix synthesis (Liu et al., 2010; Ruiz et al., 2010)and by other traditional methods (Fawaz and Mittal, 2014).

### 3.1 Polymer nanocomposites as antibacterial agents

Polymer nanocomposites have demonstrated a potential efficacy against bacterial infections among various nanocomposites. Numerous nanoparticles have been found to have a potent antibacterial impact when in combination with a polymer matrix. Graphene and copper-incorporated polymer nanocomposites are the subject of greater research out of all the known polymer nanocomposites, as these materials also exhibit higher antibacterial properties and carbon-based nanocomposites are used to enhance the property of the polymers.

#### 3.1.1 Graphene-incorporated polymers

Poly (N-vinyl carbazole) based materials are used majorly in biological applications due to their antimicrobial properties. In this regard, researchers (Mejías Carpio et al., 2012) developed a PVK-GO nanocomposite that inhibited *E. coli* biofilm by 93.6% but only 67.7% suppression was seen with GO samples. It has also been demonstrated that PVK-GO, with 97% lower concentrations has stronger antibacterial effects than GO alone. The antibacterial efficacy against *E.coli*, *Cupriavidus metallidurans, B.subtilis,* and *Rhodococcus opacus* could be attributed to reduction in metabolic activity of cells due to encapsulation. Poly (vinyl alcohol) (PVA), is a biodegradable, water-soluble polymer and biocompatible with low cytotoxicity, used for various biological applications. Methylated melamine grafted polyvinyl benzyl chloride was used as an additive in PVA and graphene nanosheets were used to reinforce the mechanical strength (Cao et al., 2015). The results obtained showed that the films exhibit strong antimicrobial effects against both *S. aureus* (99.7%) and *E. coli* (97.1%) with an increased activity recorded in the highest graphene-loaded region. PLA (Polylactic acid) is one of the most used synthetic biodegradable polymers for medical applications. It has been combined with different G-based nanomaterials and their mixtures, resulting in nanocomposites with higher antibacterial activity against *S.aureus* and *E.coli*. Compared with PLA, the addition of GO inhibited the proliferation of bacteria and cell adhesion of *S. aureus* and *E. coli* (Arriagada et al., 2018).

Certain G-based hydrogels are used in drug delivery applications due to their high potential. Researchers developed poly (acrylamide-*co-*acrylic acid) hybrid hydrogels with poly aspartic acid penetrating polymer networks with graphene, and metal nanoparticles (Ag, CuO, and ZnO) (Sattari et al., 2018). The antibacterial efficacy and drug delivery application were investigated with curcumin. The results demonstrated that loading capacity, the rate of release of curcumin, and the antibacterial activity of the hydrogel were dependent on the type of metal nanoparticle. The hydrogels containing Ag NPs exhibited higher antimicrobial activity against *E. coli* than those with embedded ZnO and CuO NPs. Further, the impregnation of curcumin was seen to enhance the activity of *E.coli.* The hydrogel was highly efficient against Gram-negative bacteria due to the stronger electrostatic interactions between the metal ion and bacteria.

#### 3.1.2 Silver incorporated polymers

As mentioned earlier AgNPs contribute to the activity of nanocomposites against pathogens. Chitosan capped on silver nanoparticles was identified to exhibit antimicrobial activity against certain bacterial species including *E. faecalis* (Raza et al., 2021). Further, coating these nanoparticles with chitosan extended the stability of the AgNPs (Cardoso et al., 2014). In addition to chitosan, AgNPs have also been stabilized using type I collagen to build a nanomaterial. Antibacterial activity was observed against *S.aureus and E.coli* without exhibiting any toxicity to the cells.

A thermochemical reduction of Ag^+^ ions in polymer PLA-AgPalm-chitosan films was used for the synthesis of an antimicrobial and antiviral nanocomposite. The synthesized nanocomposites exhibited strong antimicrobial activity against *E. coli* and *S.aureus* (Demchenko et al., 2022b). It was shown that silver-containing nanocomposites formed *in situ* demonstrate antimicrobial activity against gram-positive bacterium *S. aureus*, gram-negative bacteria *E. coli*, *P. aeruginosa*, and *C. albicans*, where the activity of the samples increases with increasing nanoparticle concentration (Demchenko et al., 2022a). Investigations into the antibacterial properties of silver/poly (lactic acid) nanocomposite (Ag/PLA-NC) films were conducted while silver nanoparticles (Ag-NPs) were chemically reduced into biodegradable PLA in a diphase solvent. Gram-positive (*S. aureus*) and Gram-negative (*E. coli* and *Vibrio parahaemolyticus*) bacteria were used to test the Ag/PLA-NC films’ antibacterial properties. The findings showed that when the amount of Ag-NPs in the PLA increased, the antibacterial activity of Ag/PLA-NC films increased (Shameli et al., 2010). AgNPs incorporated into cationic polymer Poly [2-(tert-butylaminoethyl) methacrylate] nanofibers also exhibited potential activity against *E.coli* and *S.aureus.* Yet another cationic polymer polyethyleneimine in combination with poly (glycidyl methacrylate) and AgNPs-based multifunctional nanocomposite was synthesized to detect and kill Gram-negative bacteria, paving the way to creating smart biomaterials with high selectivity (Gong et al., 2019).

#### 3.1.3 Copper-incorporated polymers

Copper nanoparticles act as reservoirs for the controlled release of copper ions. The antimicrobial activity increases on incorporating the copper nanoparticles with polymers. Since they are capable of the long-term release of ions, thus prolonging the antibacterial effect (Cioffi et al., 2005) and due to the synergy between copper and polymer (Bogdanović et al., 2015). Same as graphene, shape, and size are the factors controlling the activity of copper nanocomposites. In a study, antibacterial activity against *S.aureus* and *P.aeruginosa* is observed to be much higher on using copper polypropylene-nanocomposites than silver polypropylene-nanocomposites. Through possibly irreversible damage to the bacteria’s cell wall, interaction between the NC surface and the pathogen enhances the effective killing of the bacteria (España-Sánchez et al., 2014).

Chitosan polymer, copper nanoparticles were synthesized using stabilizing agent iodine. The antibacterial studies were carried out on *E. coli* and *B.cereus.* The antimicrobial activity was higher against *E. coli* (Mallick et al., 2012). CS-Cu NP were found to be attached to the cell wall leading irreversible damage to the membrane and eventually cell death.

The copper (II) sulfate polyacrylonitrile (PAN) composite nanofibers were incorporated with copper oxide and copper hydroxide nanowires, copper nanoparticles and were tested for their antimicrobial activity. The result showed that all copper composite nanofibers exhibited good antimicrobial activity against *E.coli* and *B.subtilis*. Cu-PAN nanoparticles exhibited the least antibacterial activity. The result of the antibacterial assay suggested that the activity depends on the size and shape of the nanoparticles and the sharp edges are considered for higher antibacterial activity of nanowires. The bioactivity was contributed by the release of soluble copper, including Cu^2+,^ however the exact mechanism remains unclear (Phan et al., 2020).

#### 3.1.4 Other polymers

Polyethylene glycol (PEG) and poly (lactide-co-glycolide) (PLGA) are biocompatible polymers approved by the U.S. Food and Drug Administration (Deepika et al., 2020). PEG–PLGA NPs were loaded with natural (rutin) and synthetic (benzamide) drug for a slow and prolonged release of drugs. The PEG–PLGA NPs exhibited antibiofilm activity against drug resistant *S. aureus* and *P. aeruginosa*. The formulation prevented the attachment of bacteria to the substrate due to the biofilm disruption. Cationic acrylate copolyvidone–iodine NPs showed no bacterial growth and demonstrated dose dependent effect. Nanocomposite maintained antibacterial effects for 11 days and it has been reported that the growth inhibition of *S. aureus* was lower than *E. coli* (Li et al., 2020).

Mannose-functionalized chitosan nanocomposites are found as good antibacterial agents against *L. monocytogenes*, *S. aureus, E. coli* and *P. aeruginosa.* Due to the interaction with the bacterial membrane lectins, the mannose functionalization greatly reduced bacterial growth, and this growth suppression was more pronounced in Gram-negative than in Gram-positive bacteria. Compared to unmodified CS NPs, mannose functionalized nanocomposites showed the most antibiofilm ability, especially against *E.coli* and *P.aeruginosa* (Ejaz et al., 2020). Cationic derivatives of betaine NPs have anti-*S.aureus* and anti-*E.coli* action. The antibacterial activity of NPs is higher than that of several other polymer nanocomposites. Zeta potential and NPs size affect antibacterial action. The smaller size and higher zeta potential value boost the antibacterial activity (Kritchenkov et al., 2020).

## 4 Phytochemical nanocomposite

The usage of chemicals as reducing agents may account for various biological risks. To overcome the scenario, serious efforts have been taken to develop an eco-friendly synthesis of nanoparticles (Ahmed et al., 2016). Plants have been used since ancient times for various therapeutic activities due to their high potential and notably phytochemicals have gained attention in recent years. The synthesis of different kinds of nanoparticles from plant derivatives can be an effective strategy to combat antibiotic-resistant bacteria. Being eco-friendly, the process of synthesis does not harm the environment majestically. Phytochemicals are majorly used as capping and reducing agents. Much research has been undertaken to study the characteristics and the mechanism of action of the phytomolecules on synthesizing nanoparticles and their antimicrobial activity. Other applications of phyto-nanoparticles include medication administration, imaging, sensing, and catalysis. When these nanoparticles contain more than one type of material, such as phytomolecules with metals or polymers, they may be referred to as nanocomposites.

The classification of a substance as a nanocomposite can be adaptable, influenced by factors like the proportions of various constituents, structure of the components, and the application of the material. If phytomolecule-based nanoparticles encompass separate elements at the nanoscale, they might rightfully be termed nanocomposites. Nonetheless, accurately describing the nanoparticles necessitates careful consideration of their unique traits and composition.

### 4.1 Antimicrobial activity of phytochemicals

Among metal nanocomposites, silver nanocomposites have been studied extensively for their potential activity. The green synthesis of AgNPs have gained attention due to their high surface to volume ratio and high antibacterial activity (Kalangadan et al., 2022). In a study by Kanniah *et al.*, *Piper nigrum* seed extracts were used for bio-reduction synthesis of AgNPs and formulation of silver-based chitosan nanocomposite (Kanniah et al., 2021). It was observed that Ag-based chitosan nanocomposite exhibited antibacterial activity against *B.subtilus* and *E.coli*. The leaf extract of *Senna alata* was also employed as a reducing agent to synthesis AgNPs and was impregnated into the agar to prepare the composite (Kalangadan et al., 2022). The composite exhibited bactericidal activity against *C. albicans, E. faecalis, S.aureus,* and *E. coli* by permeabilizing the cell membrane and deactivating the enzymes.

In a study, AgNPs are formed with the extracts from lichen, inhibited the growth of *P. aeruginosa* and MRSA. The highest antibacterial activity of AgNPs was exhibited in *P.aeruginosa*, followed by MRSA (Alqahtani et al., 2020).The AgNPs were synthesized using aqueous extract of *Tropaeolum majus*. The existence of a variety of reducing phytochemicals including tannins, terpenoids, flavonoids, and cardiac glycosides were identified by preliminary screening. The aqueous and methanolic extract retarted the growth of *B.subtilis* and their synthesized AgNPs were effective against *S.aureus* (Bawazeer et al., 2021).

*Cinnamomum tamala* leaf extract was used to synthesize the silver nanoparticles. The antibacterial effects of the synthesized nanoparticles were studied and observed that the MIC and MBC of AgNPs against *E. coli* were 12.5 and 15 μg/mL and in *S. aureus* they were 10 and 50 μg/mL, and in *K. pneumonia*, both values were 12.5 μg/mL. It was concluded that to kill the bacteria, 8 h of treatment with silver nanocomposites was sufficient as evidenced by time kill assay (Dash et al., 2020). Tyavambiza *et al* was the first to synthesize silver nanoparticles from *Cotyledon orbiculata*. The antimicrobial activities of the nanoparticles against pathogens *S. aureus*, *S. epidermidis*, MRSA, *P. aeruginosa* and *C. albicans* was evaluated, among which the highest antimicrobial activity was against *P. aeruginosa* followed by MRSA*, S. epidermidis, S. aureus,* and *C. albicans*. Cotyledon-AgNPs had greater antibacterial activity than the two employed controls against MRSA and *P. aeruginosa* (Tyavambiza et al., 2021).

The biosynthetic process extended beyond the production of AgNPs. A variety of metal nanoparticles were also generated utilizing the environmentally friendly approach. Gold and zinc oxide nanoparticles, along with nanocomposite of gold and zinc oxide, were synthesized by employing natural fibers extracted from *Phoenix dactylifera L*. seeds to act as both capping and stabilizing agents (Chamkouri et al., 2023). Observations indicated that greater antibacterial efficacy against *S. aureus* and *K. pneumoniae* was achieved when the metals were applied as nanocomposites, surpassing their individual applications, while simultaneously exhibiting low toxicity to cells. In a study conducted by Asghar et al., metal nanocomposites comprising silver, copper, and iron were synthesized utilizing extracts from *Syzygium cumini* leaves. Among these, the silver nanoparticles exhibited remarkable antimicrobial properties against antibiotic-resistant *S. aureus* strains, as well as the fungal species *Aspergillus flavus* and *A. parasiticus* (Asghar et al., 2020). Abbas et al., investigated the utilization of plant extract from Cactus *Opuntia monacantha* to biologically synthesize MgO and MgO/CuO nanoparticles. The plant’s methanolic extract was found to contain flavonoids and terpenoids, both of which played a role in the nanoparticle formation, as confirmed through a phytochemical assay. The resulting nanoparticles, along with their combined effects with antibiotics, exhibited impressive antimicrobial properties against *C.albicans* and *B.subtilis* (Abbas et al., 2022). In a study raconducted by Krol et al., ZnO nanoparticles were synthesized using an extract from *Medicago sativa* leaves. Utilizing modern instrumental techniques, the presence of polyphenols, flavonoids, and proteins in the extract was revealed. The antimicrobial potential of the ZnO nanoparticles was assessed against various organisms, including *S.epidermidis*, *Lactococcus lactis*, *Lactobacillus casei, C.albicans*, and *S.cerevisiae*. The ZnO nanoparticles exhibited antimicrobial activity against all evaluated microorganisms, with MIC values for bio-ZnO Nps ranging from 0.58 to 9.31 μg/mL (Król et al., 2019).

Copper Oxide/Carbon (CuO/C) nanocomposites were synthesized through the utilization of leaf extract from *Adhatoda vasica*. The leaf extract was employed for the creation of nanocomposites, acting as both a capping agent and a source of reduction and carbon. Upon examination, the nanocomposites demonstrated significant inhibition of bacterial strains including *K. pneumoniae, P. aeruginosa, E. coli,* and *S. aureus*. Notably, the nanocomposites exhibited inhibition against *A.niger* and *C.albicans*, with the MIC value against *K. pneumoniae* calculated at 0.791 mg/mL and against *C. albicans* calculated at 0.873 mg/mL (Bhavyasree and Xavier, 2020).

An investigation conducted by Ssekatawa et al. evaluated the effectiveness of copper oxide nanoparticles synthesized using extracts from *Camellia sinensis* and *Prunus africana* bark against carbapenem-resistant bacteria. Dynamic Light Scattering analysis revealed a net zeta potential of +12.5 mV. The CuONPs synthesized through this phyto-synthesis exhibited notably extensive growth inhibiting zones, showcasing robust antibacterial potency against MRSA, carbapenem-resistant *E. coli,* and *K. pneumoniae* at low MIC (Ssekatawa et al., 2022).

The combination of copper metals with aqueous bark extract from *Thespesia populnea* demonstrates effective action against microbes responsible for skin infections. The antimicrobial potential of CuONPs is particularly potent against bacteria such as *S. aureus*, *Streptococcus pyogenes,* and *P. aeruginosa*, as well as fungal strains like *Trichophyton rubrum* and *C.albicans*. Notably, among various microbial strains, the fungus Trichophyton rubrum exhibited a pronounced zone of inhibition, signifying a significant inhibitory effect (Narayanan et al., 2022).

Cross-linked polymeric nanoparticles, formed by utilizing methyl methacrylate and Triethylene glycol dimethacrylate copolymers, were synthesized to function as carriers for encapsulating D-limonene, an essential compound found in citrus peel. Subsequently, these nanoparticles underwent antimicrobial evaluation targeting *B.subtilis, E. coli, B.cereus,* and *S. aureus*. The results indicated that D-limonene-loaded nanoparticles exhibited inhibitory actions against all examined microorganisms. Moreover, the antimicrobial efficacy was found to correlate with the concentration of D-limonene (Andriotis et al., 2021).

**FIGURE 5:**
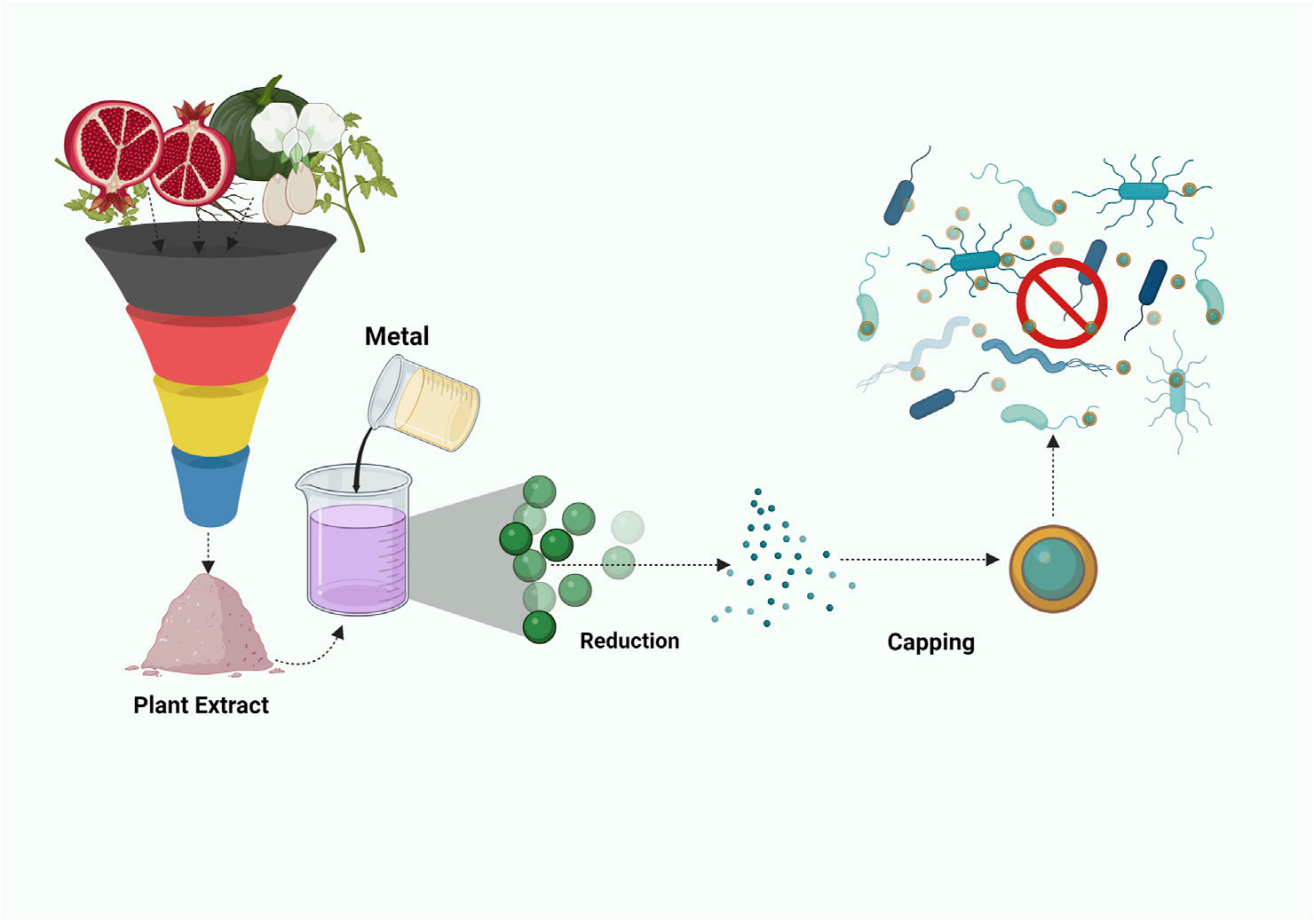
Illustration depicting the amalgamation of *Punica Granatum* and Chitosan with Gold Nanoparticles in a nanocomposite for combatting pathogens.

Hussein et al.’s study outlines the utilization of pomegranate extract (PE) from Punica granatum, in conjunction with Chitosan (CS), to function as a reducing agent for generating gold nanoparticles (Figure 5). During the incorporation of gold particles, the PE and CS collaboratively initiate the reduction process, resulting in the formation of gold nanocomposites enveloped by the CS-PE mixture. Notably, the CS-PE extract combined with gold nanoparticles demonstrated increased antimicrobial efficacy against MRSA bacteria, with a MIC of 15.6 μg/mL. While PE on its own was reported to reduce gold particle size by 50% and the precise mechanism of action remains unclear. Nevertheless, it can be postulated that PE’s multifaceted constituents might contribute to its potential as an antimicrobial agent. The potential mechanism of action could involve the disruption of the cell wall through the creation of perforations and indentations, which subsequently results in the release of cellular contents, ultimately causing cell death. In this process, chitosan likely plays a pivotal role, as highlighted in the work by Kalaivani et al. Additionally, chitosan might hinder mRNA and protein synthesis by binding to bacterial DNA, which ultimately leads to the death of the cell (Friedman et al., 2013).

## 5 Laboratory to reality

Despite the promising potential of various nanocomposites in the treatment of infectious diseases, their clinical-scale implementation faces persistent challenges. Nanotoxicology, a specialized field, addresses the toxicity of all nanomaterials, encompassing nanoparticles and nanocomposites, on living organisms. Drawbacks associated with the use of nanocomposites emerge from their synthesis through to application. Factors influencing the toxicity of nanocomposites include both exogenous (material composition, size, shape, surface modification, surface area, and charge) and endogenous (degradation) factors (Rahimi and Doostmohammadi, 2020).

During manufacturing, nanocomposites release nano and micro-scale fragments due to thermal and mechanical stress, photodegradation, and incineration. These fragments pose occupational hazards as they can be inhaled. Their pervasive nature allows them to bypass the body’s natural defenses, disrupting crucial cellular processes and contributing to a range of diseases. For instance, while TiO_2_, carbon nanotubes, and carbon nanoparticles show potential as antibacterial agents with low cytotoxicity *in vitro*, *in vivo* experiments have demonstrated lung inflammation, hyperplasia, and genotoxicity upon inhalation (Ma-Hock et al., 2009; Bonner, 2010; Han et al., 2020). Preventing undesirable particle aggregation during synthesis is a significant challenge due to high surface energy and susceptibility to oxidation, which can compromise the nanocomposites’ antibacterial properties. Achieving a uniform redispersion of nanoparticles is a complex task, carrying the risk of degradation and functional loss (Ghazzy et al., 2023). Although AgNPs have been long known for their antibacterial potency against various pathogens and non-cytotoxic to few cell lines, they have been identified to be toxic to human bronchial epithelial cells, HUVECs, red blood cells, macrophages, liver cells. The cytotoxicity was observed AgNPs with sizes (≤10 nm), dose and capping agent (Zhang et al., 2014). The AgNPs were also found to less effective against HeLa cells in comparison to HEK-293 cells, exhibiting cytotoxicity (Liu et al., 2021).

To mitigate the toxicity associated with nanocomposites, common strategies include altering the type of polymer used or employing a capping agent like PEG or chitosan (Naskar and Kim, 2019). However, comprehensive *in vivo* studies are essential to examine targeted drug delivery, nanocomposite stability within the body, leakage mechanisms, and their fate. Furthermore, a systematic investigation of the mechanism of action on drug-resistant pathogens, impact on gene expression, and metabolic pathways is imperative before considering clinical applications of nanocomposites as antibacterial agents. Finally, selecting an appropriate synthesis method, solvent, and polymer is crucial for upscaling production and eventual commercialization for therapeutic purposes (Ghazzy et al., 2023).

## 6 Conclusion and future outlooks

**FIGURE 6:**
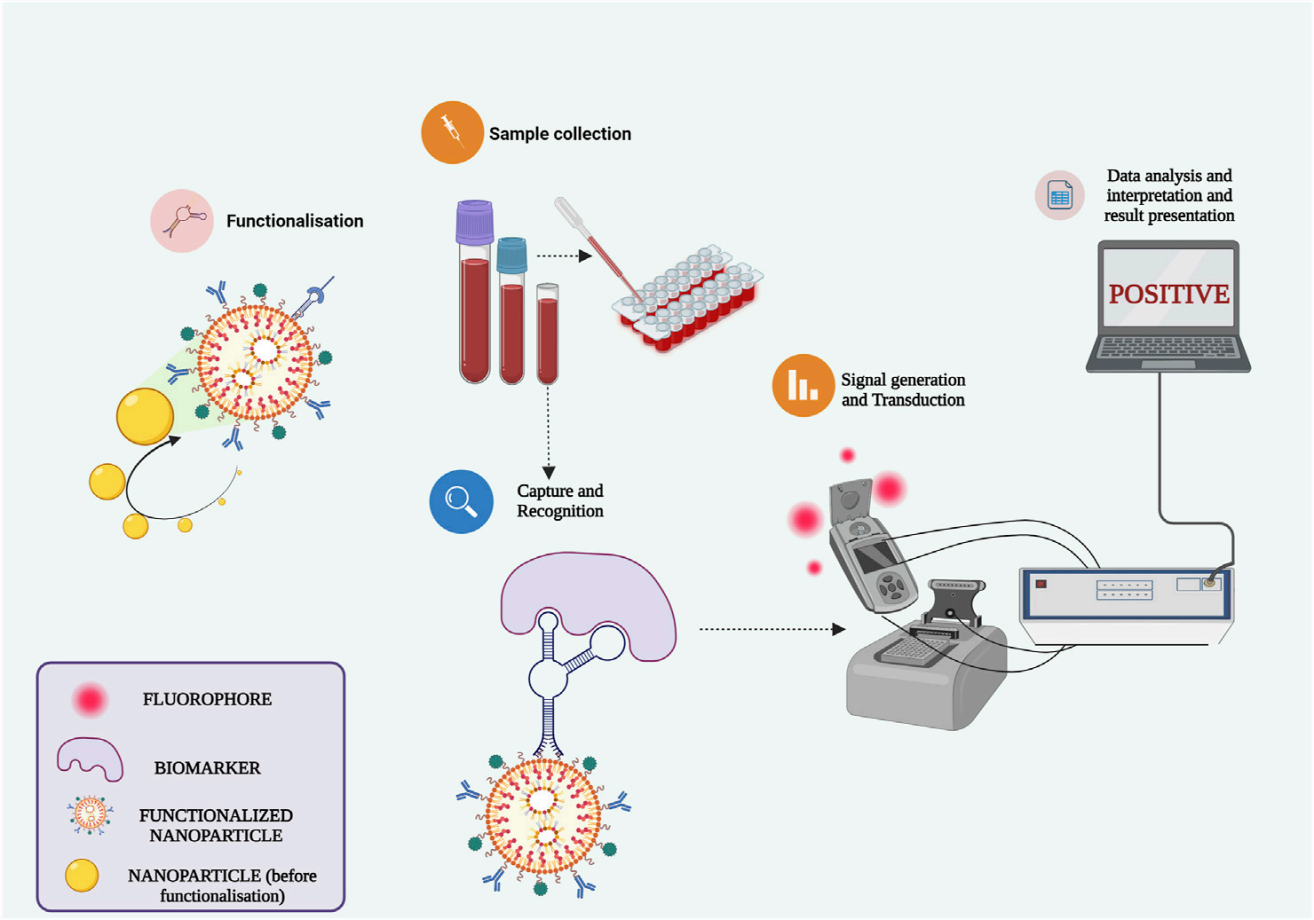
Harnessing functionalized nanocomposites for advancing biosensors and point-of-care D.evices.

The review encompasses the synthesis of various nanocomposites, combining metals and phytomolecules, aimed at combating infections and tackling multidrug-resistant pathogens. Going beyond nanoparticles, nanocomposites represent a significant breakthrough across biotechnology, microbiology, and biomedical research. Their nanoscale dimensions confer distinctive benefits like augmented surface area and reactivity, thus holding potential to transform the medical field. With the escalating threat of infectious diseases and the rise of hospital and community-acquired infections, innovative solutions are imperative. Nanocomposites emerge as promising candidates due to their remarkable properties. Exploiting their antimicrobial potential could potentially lead to breakthroughs in addressing antimicrobial resistance. Consequently, nanocomposites can evolve into biosensors, smart biomaterials for bacterial eradication, and customized drug delivery systems, enhancing treatment outcomes and mitigating antibiotic resistance risks (Figure 6). However, it’s crucial to highlight that while the promise of nanocomposites is captivating, challenges pertaining to toxicity, safety, and scalability must be systematically addressed prior to their practical clinical application. Thorough research is indispensable to fully comprehend their advantages and limitations. In essence, the integration of nanotechnology into infectious disease research marks a transformative shift, offering optimism for more efficacious therapeutic approaches. As we delve deeper into the intricate interplay between nanocomposites and pathogens, we move closer to a future where these advanced materials could substantially reshape the landscape of infectious disease and nosocomial infection treatment.
